# Multiple sclerosis: clinical profiling and data collection as prerequisite for personalized medicine approach

**DOI:** 10.1186/s12883-016-0639-7

**Published:** 2016-08-02

**Authors:** Tjalf Ziemssen, Raimar Kern, Katja Thomas

**Affiliations:** MS Center Dresden, Center of Clinical Neuroscience, Department of Neurology, University Hospital Carl Gustav Carus, Dresden University of Technology, Fetscherstr 74, 01307 Dresden, Germany

**Keywords:** Multiple sclerosis, Personalized medicine, Multiple Sclerosis Documentation System

## Abstract

Multiple sclerosis (MS) is a highly heterogeneous disease as it can present inter-individually as well as intra-individually, with different disease phenotypes emerging during different stages in the long-term disease course. In addition to advanced immunological, genetic and magnetic resonance imaging (MRI) profiling of the patient, the clinical profiling of MS patients needs to be widely implemented in clinical practice and improved by including a greater range of relevant parameters as patient-reported outcomes. It is crucial to implement a high standard of clinical characterization of individual patients as this is key to effective long-term observation and evaluation.

To generate reliable real-world data, individual clinical data should be collected in specific MS registries and/or using intelligent software instruments as the Multiple Sclerosis Documentation System 3D. Computational analysis of biological processes will play a key role in the transition to personalized MS treatment. Major breakthroughs in the areas of bioinformatics and computational systems biology will be required to process this complex information to enable improved personalization of treatment for MS patients.

## Background

Multiple sclerosis (MS) is a neurological disease of the central nervous system that is characterized clinically both by relapses caused by inflammatory demyelination and by neurological disability caused by axonal damage [1]. Since the first MS symptoms occur early in adulthood and life expectancy is only mildly reduced, MS is considered a long-term disease that affects the patient for decades [2]. MS is a highly heterogeneous disease as it can present inter-individually as well as intra-individually, with different disease phenotypes evident in different disease stages, which is probably not only true for the etiology, pathological features and immunological findings, but also for the disease course and response to treatment [3, 4]. As current treatments for MS mainly target the early inflammatory processes, the current treatment strategy is to start treatment early to prevent neurodegeneration [5, 6]. The need to ensure proper treatment for the patient with MS, considering the various clinical and immunopathological subtypes of the disease, requires clear profiling or characterization of the individual patient, definition of clinical criteria for responsiveness and/or treatment failure and collection of real world data in MS patient care [7–9].

### Need for data collection in MS care

During this lifelong disease, a large amount of medical data accumulates with important information pertaining to medical conditions, symptoms as well as diagnostic and therapeutic measures [10]. Even with proper documentation, in individual cases the assessment of responders and non-responders to immunomodulatory therapies is very difficult. Certainly, in the absence of such documentation, assessment is impossible [8, 11–13]. If one adds the characterization of psychological symptoms (e. g. depression, fatigue) and other medical disciplines (e. g. urology, ophthalmology, neuroradiology), the necessity for complex and comprehensive documentation becomes clear [14].

With complex data comes the need for electronic data processing [15, 16]. By using suitable database systems, individual disease progressions can be documented in a standardized way, and all the data can be saved for rapid and easy retrieval in a clear format. Automatic calculations lower the threshold for a systematic application of established scales which are currently indispensable for quantifying neurological deficits [17]. Interestingly, expert recommendations now mandate the regular use of scales such as the Expanded Disability Status Scale (EDSS) or the Multiple Sclerosis Functional Composite (MSFC) [18, 19].

Despite many convincing arguments for a detailed electronic documentation of MS patients, implementation in clinical practice remains difficult [20, 21]. One factor behind slow adoption of scales is a lack of consistency in how physicians are remunerated for their time spent filling out documentation, and the lack of motivating initiatives on the part of the institutions assuming the cost of treatment [22]. Only in the Scandinavian countries and in Australia have national registries have been implemented with relevant governmental support [23–26]. These registries are set to to form the interface between the national registries and the in- and outpatient records in use in hospitals and clinics, thus making registration easy and user-friendly for the clinician with immediate payback to the individual physician and patient as a time-line based graphical display on a computer screen showing information on demographics, clinical manifestations, genetic profile, biomarkers including CSF, MRI and treatment. Development of software applications based on a structured clinical records can harvest key information for registries already underway [27, 28]. The pharmaceutical industry has developed various electronic documentation systems which, after an appropriate pilot phase, have not been further pursued [13, 29]. Looking behind the reasons for this failure, it becomes apparent that a documentation platform that is dependent upon a single pharmaceutical manufacturer is does not have widespread traction. To date, MS Base is the only independent initiative in MS that has survived transfer from a pharmaceutical company to the public domain [30].

At present, major sources of MS big data, including clinical registries, electronic health record data and administrative databases (for example, claims for services and pharmaceuticals) chiefly contain classical clinical parameters for evaluation of MS. As time moves forward, more data are available from sophisticated sources such as medical imaging, biomarkers, and other ‘omics.’ Modern technologies, including wearable devices are also able to generate data, as are internet resources such as social media web sites.

### Clinical profiling of MS patients

An increased understanding of MS and its pathology, together with general concern that these descriptors may no longer adequately reflect recently identified clinical aspects of the disease, prompted a re-examination of MS disease phenotypes [31]. Some clinical manifestations may be too subtle to detect easily no matter how frequently they are assessed [32]. Following patients closely for cognitive, visual, and other clinical changes could provide clinical evidence for disease activity, functionality and clinical disability [33, 34].

The Multiple Sclerosis Performance Test (MSPT) is a computer-based platform for precise, valid measurement of MS severity [35]. Based upon and extending the MSFC, the MSPT provides precise, quantitative data on walking speed, balance, manual dexterity, visual function, and cognitive processing speed.

**Fig. 1:**
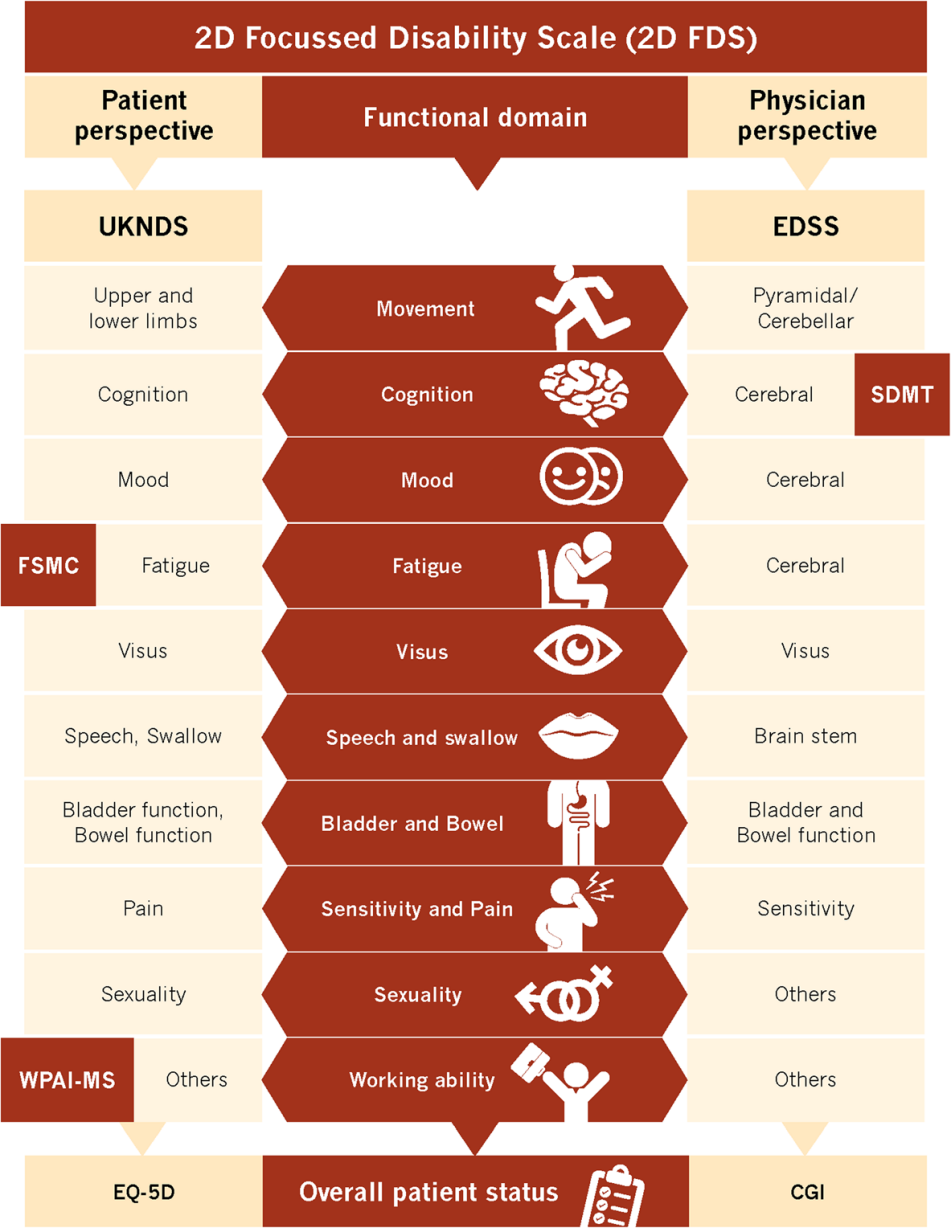
Two-dimensional functional disability scale (2D FDS) including clinical and subclinical measures representing both the patient’s and the physician’s perspectives in MSDS3D

Our software MSDS3D implements the two-dimensional functional disability scale (2D FDS), which comprises clinical and subclinical measures representing perspectives from both the patient and the physician (Fig. 1) [36]. Since deterioration not only occurs in the motor, visual, and sensory systems, this scale additionally includes cognitive changes, mood swings, fatigue, bowel and bladder function, sexual dysfunction, quality of life, as well as work productivity and activity. The overall aim is to obtain a comprehensive picture of the patient’s disease status. All the measures listed are integrated into our ‘state of the art’ evaluation of patient status.

Consensus is lacking regarding the use of patient-reported outcomes (PRO) and whether they are useful indicators of disease status [37]. Tools for the remote assessment of patient performance outside of clinical settings may prove useful in better understanding PRO –certainly, more correlative research in this area would be useful [38–42]. Ease of administration has increased with the availability of electronic PRO data collection software and web-based data entry options, allowing for immediate scoring that can be displayed for review during clinical encounters [43]. Several studies demonstrate that electronic data collection is preferred in clinical care compared to paper-based approaches [44–46]. Reproducibility of electronic data collection and responder rates are high, which reduces instances of missing data [47, 48]. Modern systems can also provide a consistent look for all content administered to patients. One of the reasons for success of this approach in clinical research has been computerized adaptive testing whereby the software selects questions on the basis of a person’s response to previously administered queries, tailoring a questionnaire with a minimal number of questions (high precision and wide-range method) [46]. Data collected using these approaches can be quite complicated to analyse and can be difficult to manage in practice,; however, first steps have been taken towards implementation into clinical practice [49]. It is worth mentioning that specific guidelines do not yet exist for the selection and application of PRO measures in MS clinical practice, and neither are these questionnaires part of a wider medical software applications.

**Fig. 2:**
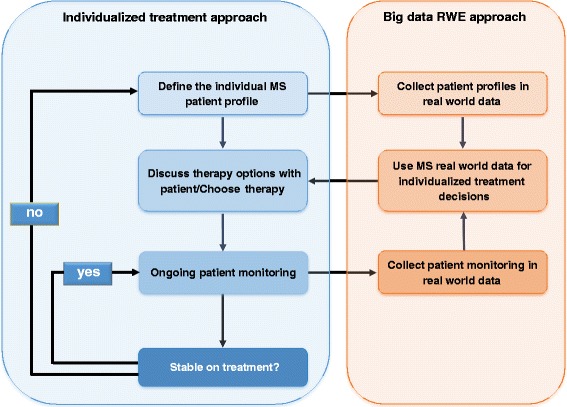
Combining individual treatment and big data RWE approach as a combined treatment approach: Individual clinical profiling is essential to characterize the individual patient in detail which is essential for individual treatment decisions. On the other side, collecting all individual patients profiles using big data approach can predict disease course and treatment responses again making personalized medicine possible

High quality clinical profiling is prerequisite to understand individual disease courses and treatment responses (Fig. 2).

### Additional parameters to be collected alongside clinical parameters

During the last decades, establishing satisfactory biomarkers for multiple sclerosis has been very difficult due to the clinical and pathophysiological complexities of the disease [50]. The emergence of immunogenetics, neuroimmunology and neuroimaging, means that detailed clinical profiling ought be complemented by assessment of potential biomarkers to inform decision making concerning strategic and individualized therapeutics [51]. However, with some exceptions, many biomarkers evaluated thus far have yet to be shown to have useful prognostic capability. The the only way to investigate their prognostic role is to combine these data on biomarkers with detailed clinical monitoring data in MS registries [25, 52]. Regardless of data type, all parameters collected as an adjunct clinical profiling depend on the quality of the medical documentation, and their quality is critical of they are to corellated with laboratory and imaging data. The availability of reliable biomarkers could radically alter our management of MS at critical phases of the disease spectrum. Identification of markers that could predict the development of MS in high-risk populations would allow for intervention strategies that may prevent evolution to established disease [53].

### MS registries as tool to collect real world data

Most studies, in particular those concerning the therapeutic effect of immunomodulatory therapies, usually occur over of 2 – 3 years of follow-up [54]. This is counter to the disease course of MS, which runs over several decades. In order to reveal long-term aspects of the disease, so-called register studies for MS patients were initiated some time ago with the goal to provide valuable information pertaining to prevalence, current therapy pattern and disease progression data [55, 56]. Such data can be collected over long periods of time, for example, more than 20 years [57]. These MS registers enable correlations of disease progression with demographic and clinical characteristics [58]. Consequently, register studies can provide data on the long-term progression of MS, wish is information urgently needed by the clinical community. Of course, MS registers have their limitations, in particular concerning the generalizability of the data [55]. A MS register only allows conclusions to be drawn for the cohort of patients documented within the scope of the register [26].

MS registers can be physician-oriented or patient-oriented with different advantages and disadvantages in each case [26, 30, 59]. The clear advantage of physician-centered registers is the detailed clinical and demographic documentation. However, the disadvantage is the high documentation effort from the physician’s side, which makes a long-term documentation of large cohorts difficult. Patient-based registers allow the collection of data from large patient numbers and they are relatively cost-effective [59]. However, the lack of medical screening as well as data validation by a physician can lead to significant errors (for example a missing MS diagnosis). Despite these problems, there are patient-centered MS registers with high response rates and 90 % or more correct MS diagnoses.

Observations of register studies are particularly valuable and clinically relevant if several register studies can confirm their findings reciprocally. However, it is important to note that real world data for different cohorts are different, and this can make correlations of this type difficult [60]. Each database is unique and usually emphasizes different features. Altogether, a lack of consistency is mostly related to quantitative and qualitative differences in data collection [54].

MS registers are the only meaningful opportunity to collect long-term data – these data cannot be collected by other means because it is not possible to track such a large number of patients over such a long time period within the scope of a clinical study. Methodologically, a prospective, standardized data collection like this overcomes the limitations in statistical power that characterize most clinical studies [56]. Nevertheless, these register databases are widely criticized for their lack of statistical power relating to poor associations and the prediction of rare events as well as their incompleteness. In addition, guaranteeing high data quality is a huge challenge, particularly when there are limited financial resources.

### MSDS 3D – combining documentation and management of MS

Since 2008 the eHealth / MSDS^3D^ project group in Dresden has been developing the patient management system Multiple Sclerosis Documentation System (MSDS) 3D, which is a new development of MSDS Klinik in practice throughout Germany [16]. Due to ever more complex diagnosis and therapy algorithms and a variety of innovations over the last years, this is an attempt to go a step further and provide not only a patient documentation system but also an electronically supported adaptive patient management system for multiple sclerosis [13, 61]. In highly specialized diseases such as MS, a specific, intelligent management system, which goes beyond a mere MS documentation, is needed by all parties involved in the treatment process [38, 62]. For prospective data collections, eg. innovative drug-specific phase IV trials, a simple documentation system that does not support the user with prospective data entry, or that does not remind him if important data are missing is not adequate [36, 63–65].

**Fig. 3:**
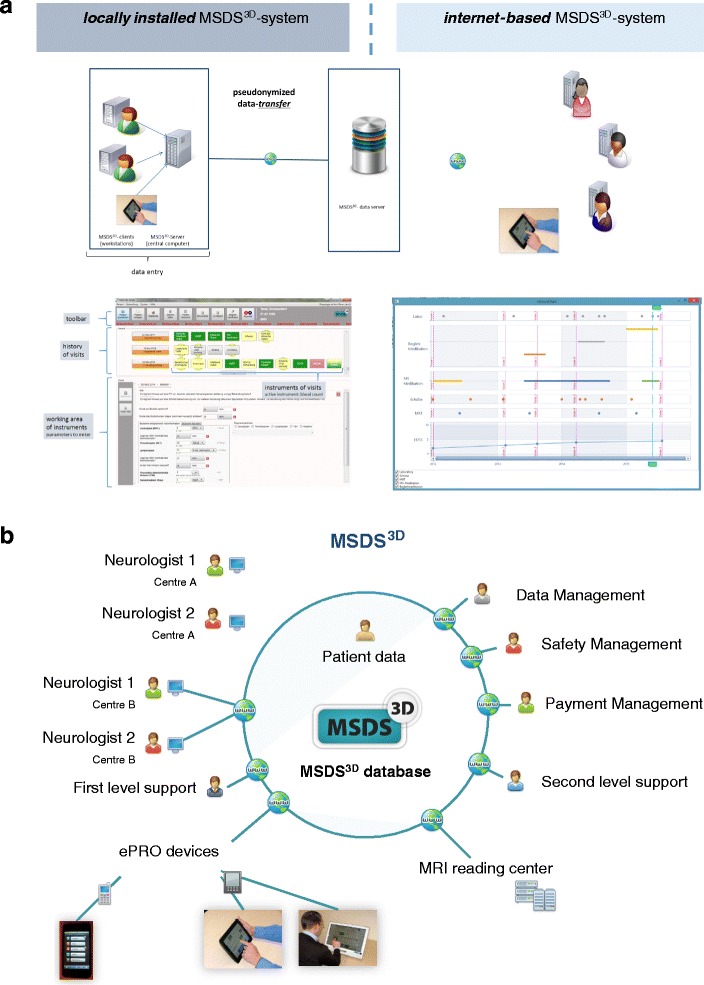
**a** MSDS3D as interdisciplinary platform to manage MS patients using the MSDS3D cloud connected with devices (**b**) MSDS3D as both local and web-based data entry system using module- and task box based technology and the MS overview

The entire process for the MSDS3D system includes the patient, nurse and physician in the system. Furthermore, the system can be used both for the collection and interpretation of patient data, and for conveying information to the patient interactively. Interaction with the patient takes place either via online multi-touch systems, such as a touchscreen or a touchpad as an interactive patient terminal, or via mobile devices such as smartphones (Fig. 3a). The interview system for the questionnaire-based data collection integrated into MSDS3D is equipped with a user interface that was specifically designed for MS patients [44, 45]. In addition, the medical staff manages the process of the interview (for example the start of the interview) and provides any necessary assistance with answering. Mobile devices are controlled by the MSDS3D system located in the local treatment center via a specific server that also controls the data flow to and from the patient. Anonymity and data protection are guaranteed by a complex process that includes an encrypted transmission. An expert advice system is included in the software to enable specialists involved in special cases to provide their opinion [64]. Neurologists who make use of clinical expert advice at any time during the study and radiologists seeking an MRI second opinion reading are asked to document these requests.

MSDS3D is a module-based, web-based or locally-installed software which is connected to a single central database (Fig. 3b). Different modules for the various MS treatments are implemented in the software to assist drug-specific management and to collect drug-specific observational data.

For each treatment, the individual MSDS3D module allows for standardized documentation and visualization of appointment schedules and the patient examinations by structuring the information on a vertical timeline with horizontally arranged tasks that are displayed as tick boxes. Each tick box related to a parameter that must be collected at a given time point. The corresponding data input menu can be opened directly from these tick boxes, and additional procedures can be added to a selected visit. Above the task box that documents the current visit of the patient, the next appointment is displayed with the planned timeframe; below this, the user can scroll through the historical completed visits. The respective tasks that have to be implemented (such as EDSS, patient interview) are displayed in by way of separate instruments [13]. Different color codes assist in monitoring the status of each task box: Green color indicates that a task has been completed by the MS nurse (e.g. patient questionnaire, cognitive tests) or the treating neurologist (e.g. EDSS, adverse effects). When all tasks of a visit have been completed, the visit is set as ‘approved’.

All data management processes will be overseen by an external data management team, which is extremely important to improve data quality and validity. Additionally, a toolbar allows for the integration of administrative features (such as creating a patient record, registering a patient for an examination) and of evaluation mechanisms into the patient management system MSDS3D. Data can be exported to various databases, an internal query process is part of MSDS3D.

An important part of MSDS3D is the safety reporting and monitoring procedures. As MSDS3D is used for phase IV studies, an easy-to-use safety reporting system is included, which is not generally seen in other observational registry initiatives.

### Clinical profiling of MS patients and data collection for MS big data

**Fig. 4:**
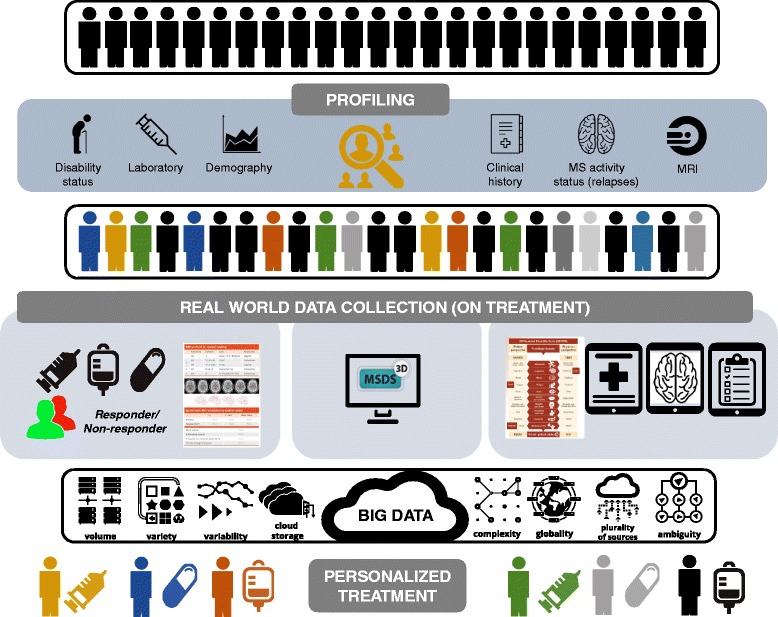
Profiling patients and collecting data. As part of the personalized medicine approach, MS patients should be characterized by a detailed clinical profiling which is the base of all long-term documentation. All patients should be documented during the course of the disease to understand treatment response, document relapses and disease progression as well as quantitative inflammatory and neurodegenerative magnetic resonance imaging (MRI) markers. Physician- and patient-centered parameters should be collected by intelligent software systems as MSDS software. MS big data can be used to analyze treatment response patterns and allow personalized treatments. Therefore, the available information (big data MS) of all included MS patients can assist in complex treatment decisions in future

All these technological approaches can be extremely useful to clinicians, providing a global, easy-to-access perspective of the disease that adds value to clinical practice: they can provide graphical representations of patients’ course, they allow benchmarking, they help to create better decision strategies and, since they collect data from large populations, they can also be used for a large number of studies (e.g. comparative drug effectiveness, pregnancy exposure outcomes, safety registries and long-term disease trends) In this way, computerized MS registries are set to form part of the future MS cloud computing and big data (Fig. 4).

### Big data analysis in MS: challenges and problems

Computational analysis of biological processes plays a key role in the transition to personalized MS treatment. According Miller et al. the following specific contributions of information technology to tailored therapy are crucial:

1.  the ability to integrate genomic, molecular, and epigenetic data about each individual patient in a unified framework,
2.  the capability to effectively analyze this information using complex queries and data mining methods, and
3.  to apply computational procedures that predict the patient’s response to treatment based on his or her genomic make-up, epigenetic tendencies, and environmental data [9].

Big data analysis in MS raises several methodological issues that need to be addressed to succeed in clinical practice. Major points include the limitations of observational data, poor data quality, data inconsistency, poor data stability, patient privacy, patient consent, and other potential legal barriers. Many data sources have significant quality limitations. The results can be valid, stable, and clinically useful only if high-quality clinical data are inputted. A related issue is the lack of documentation standards in MS care, which exacerbates the inconsistency in different data sources. The potential threats to the validity of real world data from observational studies include both unmeasured confounding factors and treatment selection bias. Legal and regulatory aspects (inadvertent release of private patient health­care data, inappropriate access to or use of patient data, and even the potential use of data to inappropriately ‘profile’ patients and provide differentiated health­care resources) also have a negative impact on successful implementation [66]. Last but not least, big data approaches will require clinical integration to be successful, facing the same implementation challenges as other health­care quality interventions that require integration into the clinical workflow to achieve clinical utility [67].

**Fig. 5:**
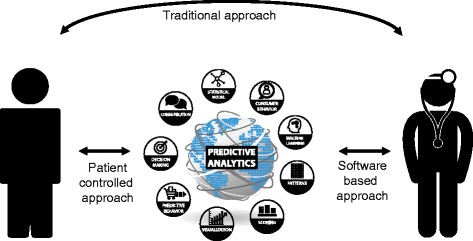
Predictive modelling in MS allowing patient controlled and software based approach in contrast to traditional approaches

Predictive models are used to inform physicians about a patient’s absolute risk of developing a defined clinical outcome and to guide patient management [68]. A predictive model includes characteristics that are statistically associated with an outcome, but not necessarily causal in the disease process. In the traditional physician-patient interaction, the patient provides the physician with the data which are necessary for evaluation. Using predictive modelling, the patient provides data to the physician who incorporates it into a decision support algorithm for diagnosis or treatment (or the patients generate and submit their own data into the predictive algorithm), allowing the physician to receive clinical insights in real time (Fig. 5).

## Conclusions

MS is multifactorial, and therefore requires the capability to model and analyze the interplay among the wide range of parameters that are involved in the onset of the disease, its progression, and the response to treatment. In addition, machine learning methods have been employed to profile MS patient gene expression response to interferon-beta [69]. To truly personalize treatment of MS, major breakthroughs in the areas of bioinformatics and biological computation systems will be required to make sense of this large and complex mix of information types.

## Abbreviations

EDSS, expanded disability status scale; MS, multiple sclerosis; MSDS, multiple sclerosis documentation system; MSFC, multiple sclerosis functional composite; MSPT, multiple sclerosis performance test; PROM, patient-reported outcomes measurements
